# Exploring soil bacterial and fungal communities in Colombian terrestrial ecosystems modulated by altitude-influenced factors

**DOI:** 10.1371/journal.pone.0312842

**Published:** 2024-12-12

**Authors:** Wendy Lorena Reyes-Ardila, Glever Alexander Vélez-Martínez, Juan Diego Duque-Zapata, Paula Andrea Rugeles-Silva, Jaime Eduardo Muñoz Flórez, Diana López-Álvarez

**Affiliations:** 1 Grupo de Investigación en Diversidad Biológica, Universidad Nacional de Colombia—Sede Palmira, Carrera, Palmira, Valle del Cauca, Colombia; 2 Facultad de Ciencias Básicas, Universidad Santiago de Cali, Calle, Cali, Colombia; Friedrich Schiller University, GERMANY

## Abstract

A bacterial (16S rRNA) and fungal (ITS rRNA) taxonomic characterization was carried out using metabarcoding along an altitudinal gradient in the western range of the Valle del Cauca, Colombia. This study encompassed Tropical Dry Forests, Andean, and Páramo ecosystems in Laguna de Sonso (900 m.a.s.l), Yotoco (1,800 m.a.s.l), Bosque del Duende (2,400 m.a.s.l), and Páramo del Duende (3,200 m.a.s.l). The physicochemical analyses revealed soils with high organic matter (>10%), non-compacted, extremely acidic pH levels (4.4) at higher altitudes, and slightly to moderately acidic pH in lower areas (5.5–6.1). 59 plant families were identified, with Araceae, Lauraceae, and Fabaceae being the most abundant. The most abundant bacterial taxonomic assignments were Acidobacteriota and Proteobacteria phyla, while for fungi, it was Ascomycota and Basidiomycota. Alpha diversity analysis showed high community diversity, whereas beta diversity reflected composition differences among locations and their heterogeneity. The most abundant functional predictions for bacteria were chemoheterotrophic activity and nitrogen cycle involvement. At the same time, for fungi, it was ecological guilds related to pathogenic activity in both animals and plants, endophytes, and epiphytic saprotrophs. The PLS-PM analysis revealed an indirect influence of altitude on microbial abundance and diversity.

## Introduction

The diversity and complexity of the soil microbiome represent a crucial yet still unknown aspect in our understanding of terrestrial ecosystem functioning [1]. This extensive microbial diversity, comprising up to a quarter of Earth’s total diversity [1, 2], raises questions about how its variability and complexity are influenced by factors associated with elevation. Altitude is one of the key environmental parameters that shape ecological niches, influencing the composition and diversity of the microbial communities present in soil ecosystems [3]. In a country of unparalleled biodiversity and vast altitudinal gradients such as Colombia, exploring the interaction between altitude and the composition of the soil microbiome opens an exciting area of research.

In Colombia, there is a broad altitudinal gradient ranging from valleys to the peaks of the Andean mountains [4], which can significantly impact the richness and diversity of bacterial and fungal communities in the soil [5]. This variation creates a natural laboratory across distinct ecosystems—Tropical dry forest, Andean forest, and páramos—each characterized by particular environmental conditions including temperature, soil properties, and plant communities [6]. By studying these ecosystems, we can evaluate hypotheses regarding the link between altitude-driven factors and the assembly of soil microbial communities, potentially revealing the mechanisms shaping microbial diversity across contrasting habitats.

Altitude induces changes in soil microbial communities, modulated by a range of environmental parameters that influence the types of organisms living in a habitat [3]. As altitude increases, temperature decreases, precipitation rises, and humidity shifts—all of which are key drivers of community composition [7]. Additionally, altitudinal gradients lead to significant changes in vegetation, with different plant communities influencing soil organic matter and root exudates, thereby affecting the bacteria and fungi closely associated with plants [8, 9]. Soil properties, including pH, organic content, and nutrient availability, are also impacted by elevation in natural ecosystems [10, 11]. Thus, a wide of interconnected factors highlights the complex influences of altitude on soil microbial diversity and ecosystems function.

Nowadays, metabarcoding has emerged as a powerful tool for studying biological diversity and understanding the ecological dynamics of organisms across various ecosystems [12, 13]. While the influence of altitudinal gradients on microbial diversity has been explored in diverse contexts, findings remain inconclusive. Some studies report a decrease in microbial diversity with increasing altitude [14, 15], while others observe no clear altitudinal effect [16]. In South America, the multiple ecosystems along the Andean mountains offer a unique opportunity to monitor the ecology of soil microorganisms influenced by various physicochemical and environmental factors, as previous studies have done [17, 18].

This study aims to characterize the taxonomic composition, diversity patterns, and functional potential of soil bacterial and fungal communities along an altitudinal gradient in Colombia. Additionally, it elucidates the relationships between altitude and environmental factors that can drive the structure of the communities in each particular ecosystem.

## Results

### Physicochemical analysis

The physicochemical analyses of soils conducted in various forested areas (Table 1) revealed significant differences in soil properties concerning altitude. An evident pattern was the decrease in pH as altitude increased. The LS (900 m.a.s.l) site exhibited a slightly acidic pH of 6.17±0.5, while Yt (1800 ma.s.l) showed a pH of 5.47±0.5; this trend continued in BD (4.4±0.2) and PDU (4.5±0.4) (2400 and 3200 ma.s.l), indicating more acidic conditions at higher altitudes. In contrast, electrical conductivity (EC) displayed an opposite behavior, with higher values in LS (2.7±0.6 dS/m) and lower in PDU (0.3±0.1 dS/m), suggesting differences in salt concentration that do not affect soil alkalinity.

**Table 1:** Physicochemical measurements of forest and páramo soil in Colombia. Mean + s.d. and Kruskal–Wallis H tests of variables used for comparisons among locations. Superscripts denote Dwass-Steel-Critchlow-Fligner pairwise comparisons between locations; means with the same letter do not differ significantly (P < 0.001 or P < 0.05).

Location	Texture	Altitude (m.a.s.l)	pH	EC (dS/m)	SOM (g/100g)	SOC (g/100g)	N (%)	P (mg/kg)	S (mg/kg)	ECEC (cmol+/Kg)	B (cmol+/Kg)
**LS**	Clayey	900	6.17±0. 5^a^	2.7±0.6^a^	3.29±0.7^a^	0.164±0^a^	0.16±0.01 ^b^	20.2±4.1^a^	272±73.7^a^	21.6±3.1^a^	0.3±0.1^a^
**Yt**	Clay loam	1,800	5.47±0.5^b^	1.34±0.9^b^	13.3±5.9^bc^	0.7±0.3^bc^	0.66±0.1 ^b^	7.9±6.5^b^	11.2±6.4^bcd^	14.7±7.4^ac^	0.2±0.1^ab^
**BD**	Clay loam	2,400	4.4±0.2^c^	0.7±0.3^b^	10.1±1.5^b^	0.5±0.1^b^	0.5±0.02 ^a^	2.9±1.2^c^	8.3±2.4^cd^	4.5±1.4^b^	0.1±0.02^b^
**PDU**	Clay loam	3,200	4.5±0.4^b^	0.3±0.1^c^	23.4±17.1^c^	1.2±0.9^c^	1.17±0.32 ^b^	6±2.9^b^	11.5±2.7^d^	9.7±2^c^	0.3±0.1^a^
H	33		25.8	26.9	21.4	20.8	20.80	25.3	21	26.2	14.1
p-value	< .001		< .001	< .001	< .001	< .001	<0.001	< .001	< .001	< .001	< .05
**Location**	**Texture**	**Altitude (m.a.s.l)**	**Ca (cmol+/Kg)**	**Mg (cmol+/Kg)**	**K (cmol+/Kg)**	**Na** **(mg/kg)**	**Fe (mg/kg)**	**Cu (mg/kg)**	**Mn (mg/kg)**	**Zn** **(mg/kg)**	**BD** **(g/cm** ^**3**^ **)**
**LS**	Clayey	900	10.1±2.4^a^	10.1±0.9^a^	0.208±0.1^a^	1.24±0.2^a^	130±48.2^a^	13.7±1.1^a^	9.92±2.4^a^	1.76±0.5^a^	0.96±0.1^a^
**Yt**	Clay loam	1,800	10±5.4^a^	3.6±3.4^b^	0.247±0.1^a^	0.278±0.4^bcd^	81.5±25.4^b^	2.71±4.4^bcd^	13.8±5.2^a^	1.7±1^a^	0.496±0.1^b^
**BD**	Clay loam	2,400	0.5±0.3^bc^	0.3±0.1^cd^	0.1±0^c^	0.1±0^cd^	269±214^a^	1.6±0.9^cd^	5.6±6.8^a^	2.8±1.4^ab^	0.7±0.1^cd^
**PDU**	Clay loam	3,200	0.5±0.4^c^	0.4±0.3^d^	0.2±0.1^a^	0.1±0^d^	805±148^a^	1.1±0.2^d^	1.7±1.4^b^	6.6±3.1^b^	0.7±0.1^d^
H	33		24.8	27.2	14.1	24.7	18.5	20.1	18.2	13.7	23.4
p-value	< .001		< .001	< .001	< .05	< .001	< .001	< .001	< .001	< .05	< .001

The SOM concentration also exhibited notable variations (Table 1). LS recorded a content of 3.29±0.7 g/100g, while PDU significantly increased to 23.4±17.1 g/100g, indicating a direct influence of altitude on organic material accumulation in soils. A similar pattern was observed for soil organic carbon (SOC), with PDU exhibiting the highest values. Likewise, the soil’s nitrogen levels followed a trend analogous to that of organic matter, where the PDU soils possessed the highest available nitrogen concentrations, in contrast to the LS soils, which recorded the lowest nitrogen levels.

Calcium (Ca) and magnesium (Mg) concentrations varied significantly across the studied soil types (Table 1). LS soils exhibited the highest levels, with Ca at 10.1 ± 2.4 cmol+/Kg and Mg at 10.1 ± 0.9 cmol+/Kg. In Yt soils, Ca remained relatively high (10.0 ± 5.4 cmol+/Kg) while Mg content was lower (3.6 ± 3.4 cmol+/Kg). PDU and BD soils showed the lowest Ca (0.5 ± 0.4 and 0.5 ± 0.3 cmol+/Kg, respectively) and Mg (0.4 ± 0.3 and 0.3 ± 0.1 cmol+/Kg, respectively) concentrations.

Regarding phosphorus (P) concentration, an inverse relationship with altitude was observed (Table 1). At the lowest elevation, LS soils exhibited the highest P content (20.2 ± 4.1 mg/kg). In contrast, P levels in PDU soils were significantly lower (6 ± 2.9 mg/kg). Iron (Fe) concentrations also displayed substantial heterogeneity across the studied zones (Table 1). Notably high Fe content was observed in BD (269 ± 214 mg/kg) and PDU (805 ± 148 mg/kg) soils.

Soil textures across the studied sites primarily ranged from clay loam, except for LS, which exhibited a clay-rich composition. These characteristics favor water and nutrient retention, partially explaining the observed variability in soil properties.

## Microbial composition

### Bacteria

Of the 37 sequenced samples, three did not meet quality standards. A total of 4,956,133 raw reads were generated, with an average of 150,185.8 ± 45,316 reads per sample. After quality control using DADA2, 2,141,322 ± 20,617.9 reads were obtained (S1 Table), corresponding to the identification of 34,751 ASVs (S2 Table). Seven samples were excluded during quality control, with three from the BD site and four from the PDU site (S2 Table).

**Fig 1:**
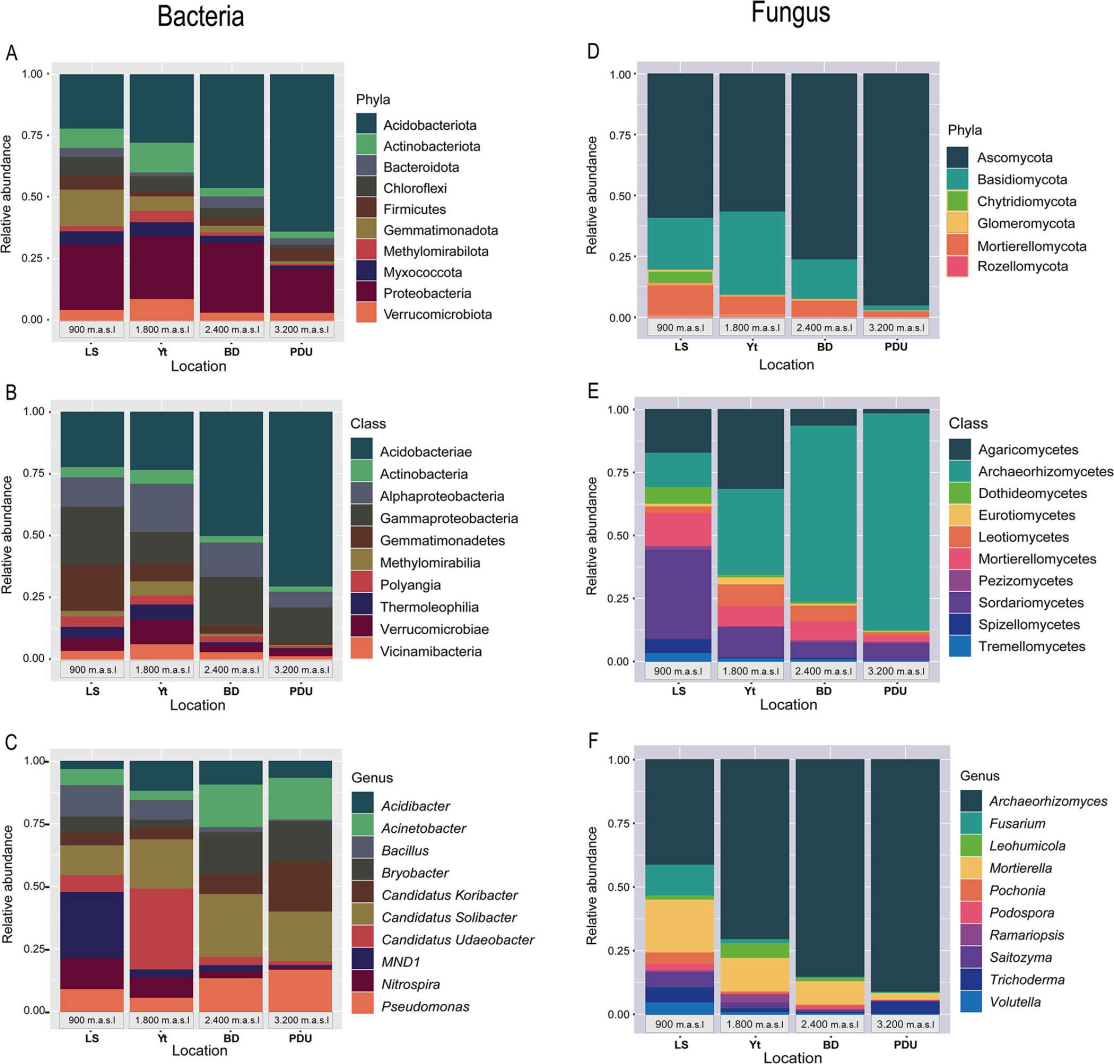
Relative abundance of microorganisms in four sampling areas along an altitudinal gradient in the Western Colombian Mountain range. The taxonomic levels for bacteria were phylum (A), class (B), and genus (C), and for fungi phylum (D), class (E), and genus (F).

In terms of taxonomy, 99.2% of the reads could be classified at different levels: phylum (99.2%), class (95.5%), order (87.6%), family (55.5%), genus (35.9%), and species (2.9%). A total of 35 phyla were identified, with ten of them representing 89.2% of the reads. The most abundant phyla were Acidobacteriota (33.1%) and Proteobacteria (23.9%), followed by Gemmatimonadota (7.3%), Actinobacteriota (6.6%), Chloroflexi (4.7%), Verrucomicrobiota (4.2%), Firmicutes (3.9%), Myxococcota (3.2%), Methylomirabilota (2.3%), and Bacteroidota (2.2%) (Fig 1).

A positive correlation was observed between the abundance of Acidobacteriota and altitude. As altitude increased, the presence of Acidobacteriota also increased. In the lowest altitude site, LS (900 m.a.s.l.), it represented 22.4% of the reads, while in the highest altitude site, PDU (3200 m.a.s.l.), its abundance reached 67.3%. The intermediate sites, Yt (1800 m.a.s.l.) and BD (2400 m.a.s.l.), recorded proportions of 28.8% and 49.5%, respectively. In contrast, no clear relationship was observed between altitude and the abundance of Proteobacteria, as its relative abundances ranged from 17.8% to 28.6% for all sites (Fig 1B).

Other bacterial phyla displayed heterogeneous abundance patterns across the altitudinal gradient, with no significant effect of altitude observed on their distribution. Actinobacteriota was most abundant in Yt (11.5%), while Gemmatimonadota and Chloroflexi were more common in LS (16.7% and 7.4%, respectively), Myxococcota was found in higher proportion in LS (4.6%), Verrucomicrobiota had its highest abundance in Yt (8.4%), Firmicutes showed similar proportions in LS and PDU (6% in both cases), and Bacteroidota was most prominent in BD (4.2%).

At the class level, the top ten most abundant groups harbored a remarkable 77% of the total classified sequences (Fig 1B). Acidobacteriae (28.7%), Gammaproteobacteria (13%), Alphaproteobacteria (10.9%), and Gemmatimonadetes (7.1%) emerged as the most prominent taxa, collectively contributing nearly half (49.7%) of the assigned reads within this category. The remaining classes, including Verrucomicrobiae, Vicinamibacteria, Actinobacteria, Thermoleophilia, Polyangia, and Methylomirabilia, exhibited lower abundance values, ranging from 3% to 6%.

In terms of the different sites, possible effects of altitude on three of the top ten bacterial classes were observed. Acidobacteriae showed an increasing trend with altitude, with a distribution of abundances in LS (23.5%), Yt (25.7%), BD (54%), and PDU (73.6%). In contrast, Gemmatimonadetes and Polyangia displayed a distinct pattern, reaching their highest abundance at the lowest altitude site, LS (20.9% and 3.4%, respectively). The remaining classes, Alphaproteobacteria, Verrucomicrobiae, Vicinamibacteria, Actinobacteria, Thermoleophilia, and Methylomirabilia, did not exhibit a clear trend of increasing or decreasing abundance with altitude. However, these classes all had their highest relative abundances at the intermediate site, Yt (1800 m.a.s.l.), with values ranging from 4.9% to 21.3%.

In the following section (Fig 1C), approximately 36% of the reads obtained in the sequencing process were successfully assigned. Among the ten most abundant genera, *Candidatus Solibacter*, *Candidatus Udaeobacter*, *Acinetobacter*, and *Pseudomonas* stood out, representing 17.6% of the total reads. When analyzing different sampling locations, distinct behavioral patterns were observed in most genera. *Nitrospira* seemed to respond to the altitudinal gradient, with higher abundance in LS and lower in PDU. On the other hand, *Candidatus Solibacter* was more abundant in BD, followed by Yt and PDU, while *Candidatus Udaeobacter* was more prevalent in Yt, and *Pseudomonas* was found in higher proportions in PDU. Other genera like *Candidatus Koribacter* and *Acidibacter* showed preferences for soils in PDU, Yt, and LS, respectively. Lastly, *Bryobacter* exhibited a notable behavior present in BD and PDU in equivalent proportions (16.1%) (Fig 1C).

Based on this composition, the analysis of differential bacterial abundance between locations revealed significant differences (S1 Fig). *Candidatus Koribacter* was significantly more abundant in LS compared to PDU, while Yt and PDU presented dominant genera like *Bryobacter* and *Bacteroides*, with the latter being more common in PDU. Furthermore, comparing BD and PDU, *Pseudomonas* stood out in BD. Similarly, between LS and BD, *Bacillus* was more frequent in LS, whereas *Acinetobacter* was more common in BD. On the other hand, between LS and Yt, *Chitinophaga* and *Bacillus* were predominant in LS.

### Fungi

For fungi, 34 samples met the quality standards and obtained 5,769,380 Illumina reads. After applying the quality control and filtering of chimeric reads, a total of 3,858,914 ± 18,728.7 reads were obtained (S1 Table). Finally, 1,974,045 reads corresponding to 17,677 ASVs were analyzed (S2 Table). However, within each component of the taxonomic characterization, sequences that were not yet identified in the UNITE database were found. As a result, the percentages of assignment decreased at each taxonomic level, obtaining the following results: Phylum: 55.7%, Class: 51.9%, Order: 44.6%, Family: 37.1%, Genus: 35.7%, and finally, Species: 7.1% (S2 Table).

Among the identified fungal phyla, the top three (Ascomycota, Basidiomycota, and Mortierellomycota) collectively accounted for 54.6% of the classified sequences (Fig 1D). These phyla seem to respond to the change in elevation, with Ascomycota being highly abundant in PDU (95.7%), while Basidiomycota showed a higher presence in Yt compared to the other locations (Fig 1D).

Five fungal classes emerged as particularly abundant across the sampling sites (Fig 1E). Archaeorhizomycetes were predominant in PDU (87.1%) and BD (72.1%), while Sordariomycetes, Agaricomycetes, and Mortierellomycetes were more abundant in LS. On the other hand, Leotiomycetes were found in higher proportions in soils of the Yt and BD localities.

At the genus level (Fig 1F), *Archaeorhizomyces* dominated across all locations, reaching remarkable abundances (LS: 37.9%, Yt: 73.2%, BD: 84.6%, PDU: 91.4%). The remaining two dominant genera, *Mortierella* and *Fusarium*, were also more abundant in LS (19.5% and 5.4% respectively). To explore differences in fungal communities between locations, a differential abundance analysis was conducted at the genus level, focusing on comparisons between LS and PDU, as well as Yt and PDU (S2 Fig). The results revealed distinct fungal compositions across the sites. In LS, *Trichoderma*, *Mortierella*, *Glutinomyces*, *Leohumicola*, and *Clavulinopsis* were among the most abundant and representative genera. Yt exhibited a different pattern, with genera like *Sclerodon*, *Pochonia*, *Clavulinopsis*, *Glutinomyces*, and *Trichoderma* being highlighted. Notably, *Archaeorhizomyces* emerged as the dominant genus in PDU in both comparisons.

### Diversity analysis

#### Alpha diversity

**Fig 2:**
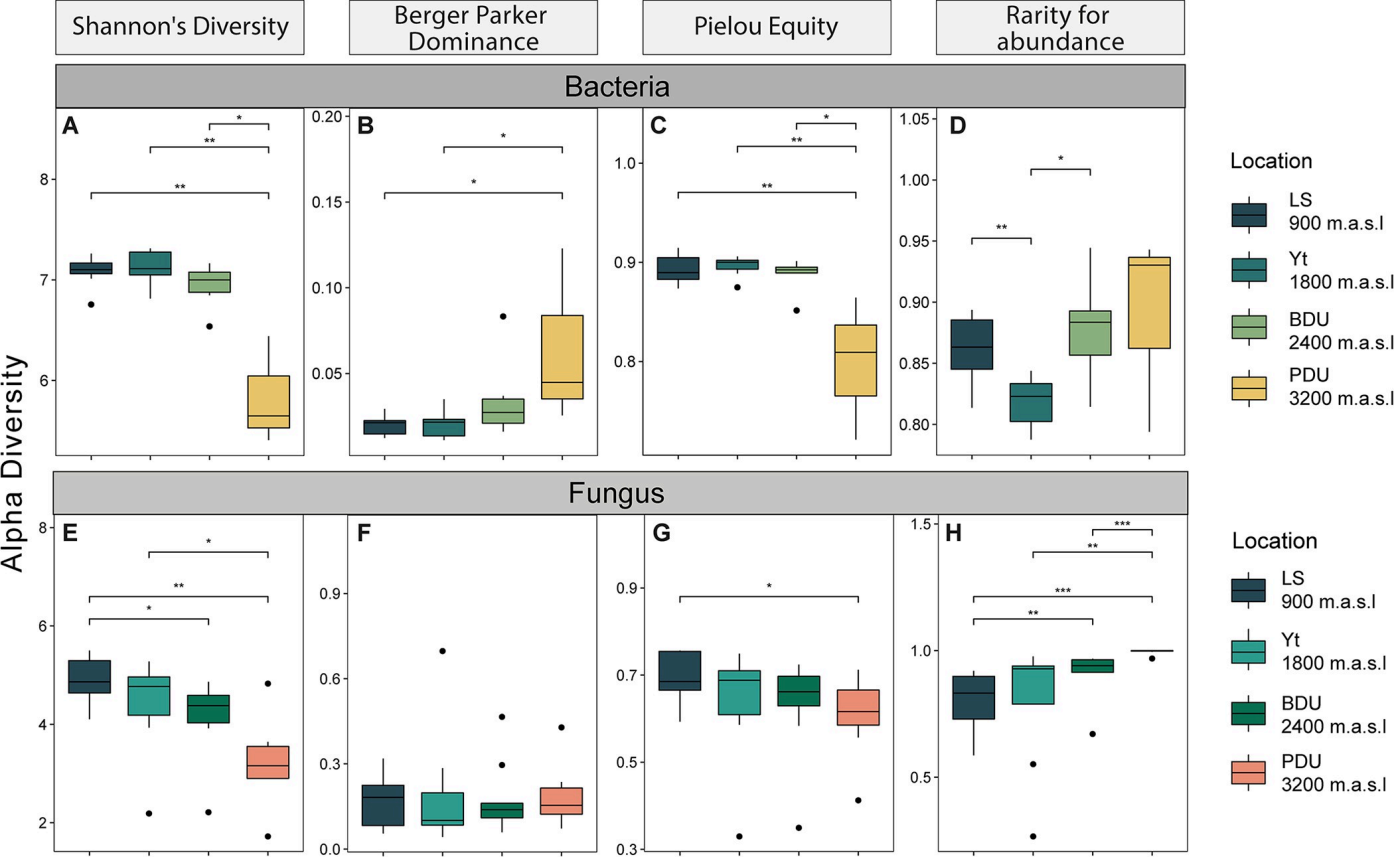
Visualization of microbial alpha diversity indices (Diversity, Dominance, Uniformity, and Rarity). Bacteria: Shannon index (A), Berger-Parker index (B), Pielou index (C), rarity by abundance index (D), and for fungi: Shannon index (E), Berger-Parker index (F), Pielou evenness index (G), rarity by abundance index (H). (*: p<0.05, **: p<0.01, ***: p<0.001).

The Shannon diversity index (Fig 2A) revealed that all locations exhibited high diversity, with values above 3. However, it is noteworthy that the PDU location showed the lowest diversity value compared to the others. When analyzing the differences between locations, statistically relevant results in terms of diversity were found. For bacteria (Fig 2A), PDU differed from LS (p<0.01) and Yt (p<0.05), while BD showed significant discrepancies with LS (p<0.05). Regarding fungi (Fig 2E), significant differences were found between PDU and BD (p<0.001), as well as Yt (p<0.01) and LS (p<0.001). Additionally, differences were observed between LS and BD (p<0.01).

**Fig 3:**
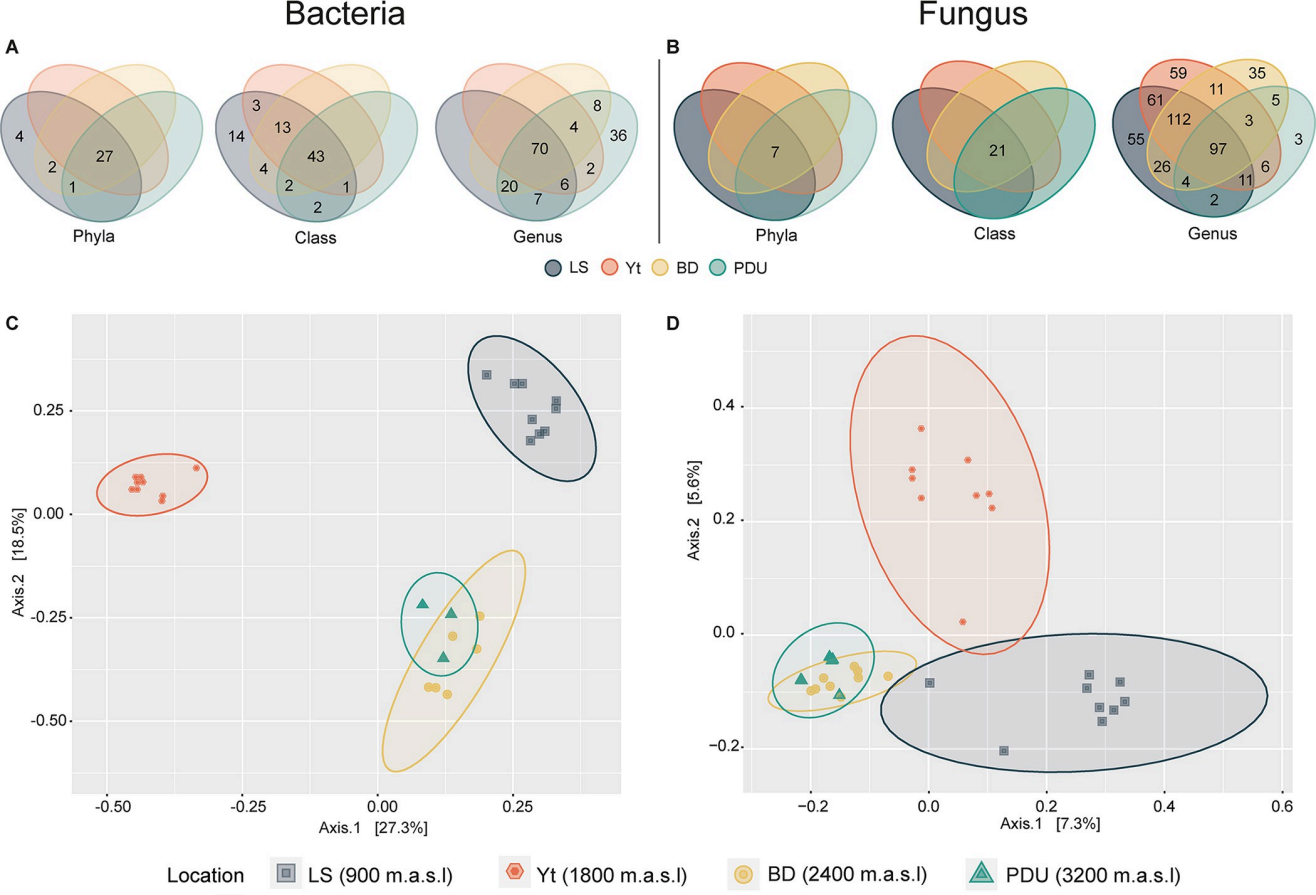
A: Venn diagrams for bacteria, B: Venn diagrams for fungi, C: PCoA in bacteria, and D: PCoA in fungi.

Dominance assessed using the Berger-Parker index (Fig 2B and 2F) did not show significant differences between locations for both bacteria and fungi; the obtained values were low, supporting the high diversity present in all samples. Concerning Pielou’s evenness index for bacteria and fungi, values close to 1 were found in all locations, suggesting a uniform distribution of species in the soil samples (Figs 2C and 3G). However, significant differences between PDU and LS (p<0.05) were observed for bacteria, as shown in Fig 2C.

Lastly, when analyzing the abundance of different and/or unique species (Figs 2D and 3H), significant differences were found between PDU and BD (p<0.001), Yt (p<0.01), and LS (p<0.001) for both bacteria and fungi. Differences between LS and BD (p<0.01) were also noted, indicating high diversity in all locations for both bacteria and fungi; however, there are significant differences in terms of diversity and composition among the different study locations.

#### Beta diversity

Venn diagram analysis of bacterial phyla revealed a core community of 27 phyla shared by all four locations (Fig 3A), suggesting a degree of taxonomic conservation across the altitudinal gradient. However, the LS location exhibited four exclusive phyla (Sva0485, Deferrisomatota, Fibrobacterota, Campilobacterota), suggesting a certain concentration of specific types of bacteria in this ecosystem place. Furthermore, a higher number of unique and/or different classes (14) were found at LS compared to the other locations (Fig 3A). This observation highlights the potential for increased bacterial adaptation to specific environmental niches within the LS ecosystem and underlines the influence of elevation on bacterial community diversity.

In fungi, a decrease in diversity was observed with increasing altitudinal gradient; however, all 7 phyla were shared among all locations, indicating some taxonomic conservation in these fungal communities. In contrast to bacteria, more pronounced differences were found at the class and genus taxonomic levels (Fig 3B). Twenty-one classes were shared at the class level, suggesting a more conserved taxonomic structure. At the genus level, similar patterns to those observed in bacteria were identified, with genera shared among all locations but also exclusive genera for each location (Yt = 59, LS = 55, BD = 35, and PDU = 3), highlighting adaptations.

PCoA analysis revealed clustering patterns in both bacterial and fungal communities, with a tendency to group by sampling location (Fig 3C and 3D). Although explained variation differed (bacteria: 45.8%, fungi: 12.9%), an interesting exception emerged. In both analyses, the BD and PDU locations clustered together despite the altitude difference. This finding suggests that geographical proximity between these two locations may be a stronger influence on the similarity of bacterial or fungi communities.

**Fig 4:**
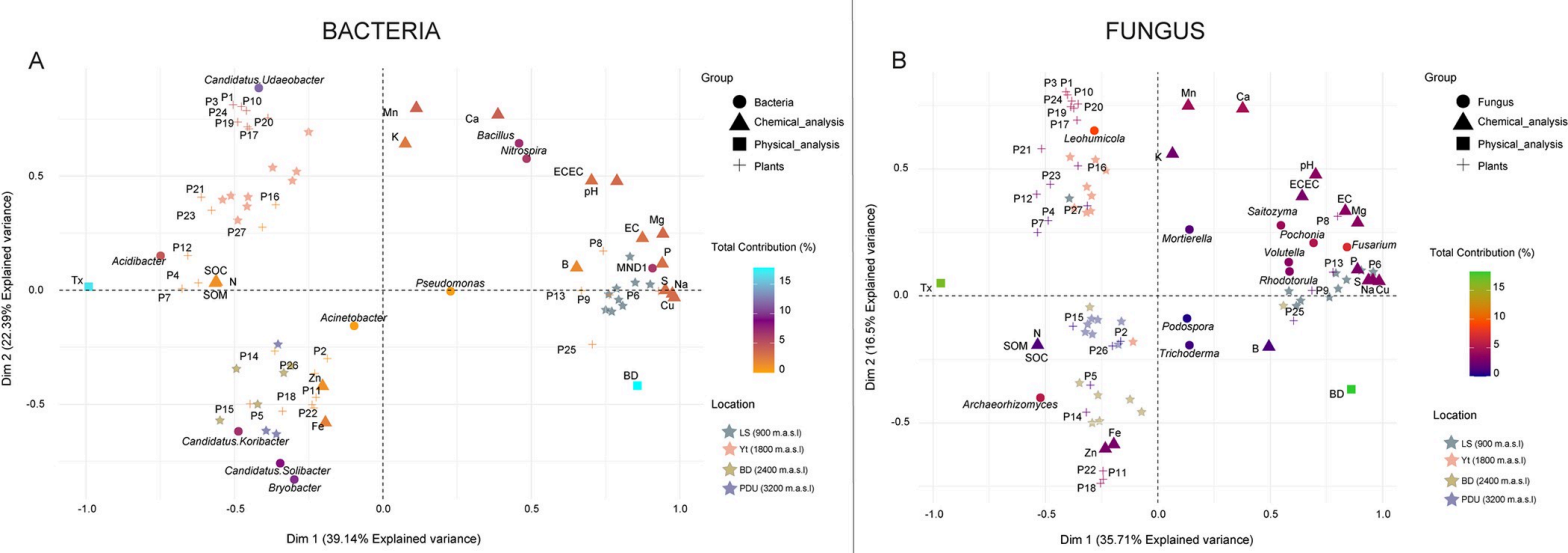
Multifactor analysis for A. Bacteria and B. Fungi. Among the observations of the variables: SPA: Soil Physical Analysis, SCA: Soil Chemical Analysis, GAB: Genera of Abundant Bacteria, GAF: Genera of Abundant Fungi, and Plants (S3 Table).

Multiple Factorial Analysis (MFA) was employed to investigate the interplay between various factors shaping bacterial and fungal communities, including soil physicochemical properties, abundance of representative microbial genera, and plant species (S3 Table) identified in the field. The first two dimensions of the MFA captured a substantial portion of the variation in these communities (39.14% and 22.39% for bacteria, 35.71% and 16.5% for fungi–Fig 4A and 4B). These findings suggest that interplay between various factors likely plays a significant role in shaping the overall microbial composition across the studied locations with similar results to those obtained in PCoA (Fig 3).

In the first quadrant, the LS location showed an association for both bacteria and fungi, with LS being related to various elements: Mg, P, S, N, Cu, EE, pH, ECEC, Ca, and Mn, as well as bacterial genera such as *Bacillus*, *Nitrospira*, and *Pseudomonas*, and fungal genera such as *Mortirella*, *Saitozyma*, *Pochonia*, *Fusarium*, *Volutella*, and *Rhodotorula*. In terms of plants, the dynamics were different: for bacteria, the plant species Casearia (P8), *Pithecellobium* (P6), and *Croton* (P13) were associated with the elements of the first quadrant, while in fungi, the plant species *Synedrella* (P9) moved from the second quadrant to the first (Fig 4). In the second quadrant, the plant species *Solanum* (P25) and *Synedrella* (P9) were highlighted, being associated with elements S, Na, Cu, and apparent density, indicating that the bacterial genus *Pseudomonas* is relevant in both quadrants 1 and 2. However, for fungi, the dynamics are different, *Solanum* (P25) was related to element B and apparent density and was associated with the fungal genera *Podospora* and *Trichoderma*. In the third quadrant, the locations BD and PDU were highlighted, being associated with high levels of Fe and Zn in the soil, with the predominant plant species in this area including *Pilea* (P2), *Guzmania* (P11), *Blechnum* (P14), *Besleria* (P15), *Chusquea* (P18), *Clusia* (P22), and *Faramea* (P26); among bacterial genera, *Bryobacter*, *Candidatus Koribacter*, and *Candidatus Solibacter* were the most abundant (Fig 4).

In contrast, the dominant fungal genus was *Archaeorhizomyces*, which was associated with the elements SOM, N, and SOC, and showed a notable affinity with one of the points of Yt. Finally, in the fourth quadrant, the predominant location is Yt, where fifteen main plant species were identified, with *Rhodospatha*, *Beilschmiedia*, and *Ocotea* being the most notable. *Leohumicola* was identified as the most representative fungal genus. In contrast, the most abundant genera in the bacterial context were *Candidatus Udaeobacter* and *Acidibacter*, which were associated with the elements SOM, N, and SOC, unlike the fungal genera.

### Functional potential

#### Bacteria

**Fig 5:**
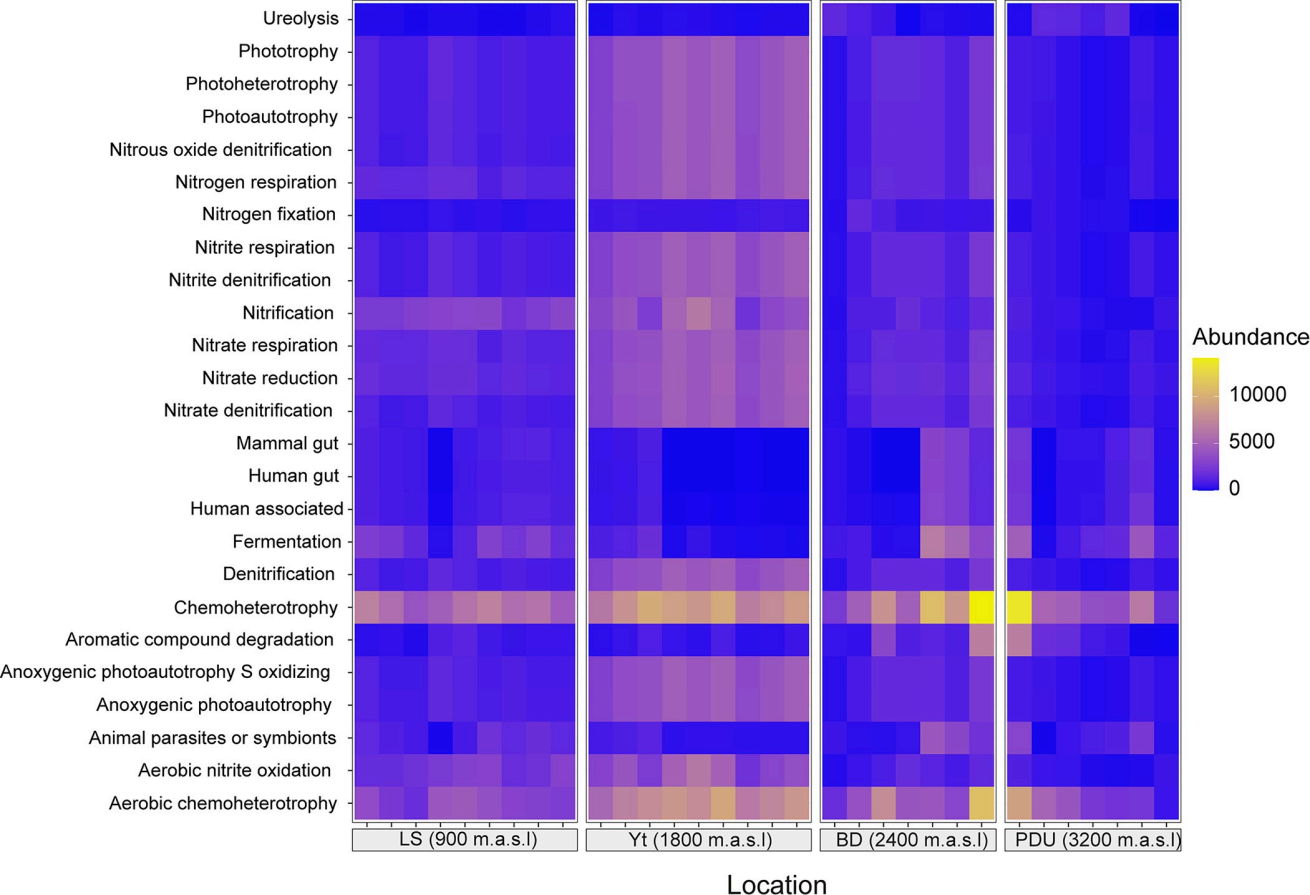
Heatmap of bacterial functional potential. Data based on ASV occurrence and their respective absolute abundances were obtained from soil samples from each location.

An analysis of bacterial functional potential was conducted using FAPROTAX, based on 16S rRNA amplicon sequencing data for 34,751 ASVs previously identified. FAPROTAX assigned functional roles to 22.8% of the ASVs, revealing 25 distinct functional categories potentially represented by these bacteria (Fig 5 and S4 Table). These findings provide valuable insights into the metabolic capabilities of the bacterial communities across the studied locations (Fig 5). The most prominent functions in all samples were chemo-heterotrophy and aerobic chemo-heterotrophy (S4 Table). Using PCA represented in S3 Fig, functions were classified into eight distinct categories. Among them, chemo-heterotrophy and functions associated with the nitrogen cycle stood out for their abundance in all locations. Specifically, in the PDU location, chemo-heterotrophy accounted for 50.7% of functions, followed by BD at 38.9%, LS at 31.2%, and Yt at 22%. Regarding nitrogen cycle functions, Yt showed the highest proportion at 54.3%, followed by LS at 44.5%.

The PCA analysis (S3 Fig) displayed the relationships between functional behavior and sampling locations. In this analysis, component 1 and component 2 explained 50.1% and 35.6% of the cumulative variance, respectively (Fig 5). Clear associations were identified between the eight functional groups and specific locations: anoxygenic photoautotrophy, photoheterotrophy, and functions linked to the nitrogen cycle were mainly related to Yt samples.

In contrast, functions related to human and mammalian gut contents, specifically those related to humans, showed a strong association with BD samples. Urea degradation was closely linked to PDU and BD locations. Notably, despite being one of the most prominent potential uses, chemo-heterotrophy did not show a specific relationship with any of the studied locations.

#### Fungi

**Fig 6:**
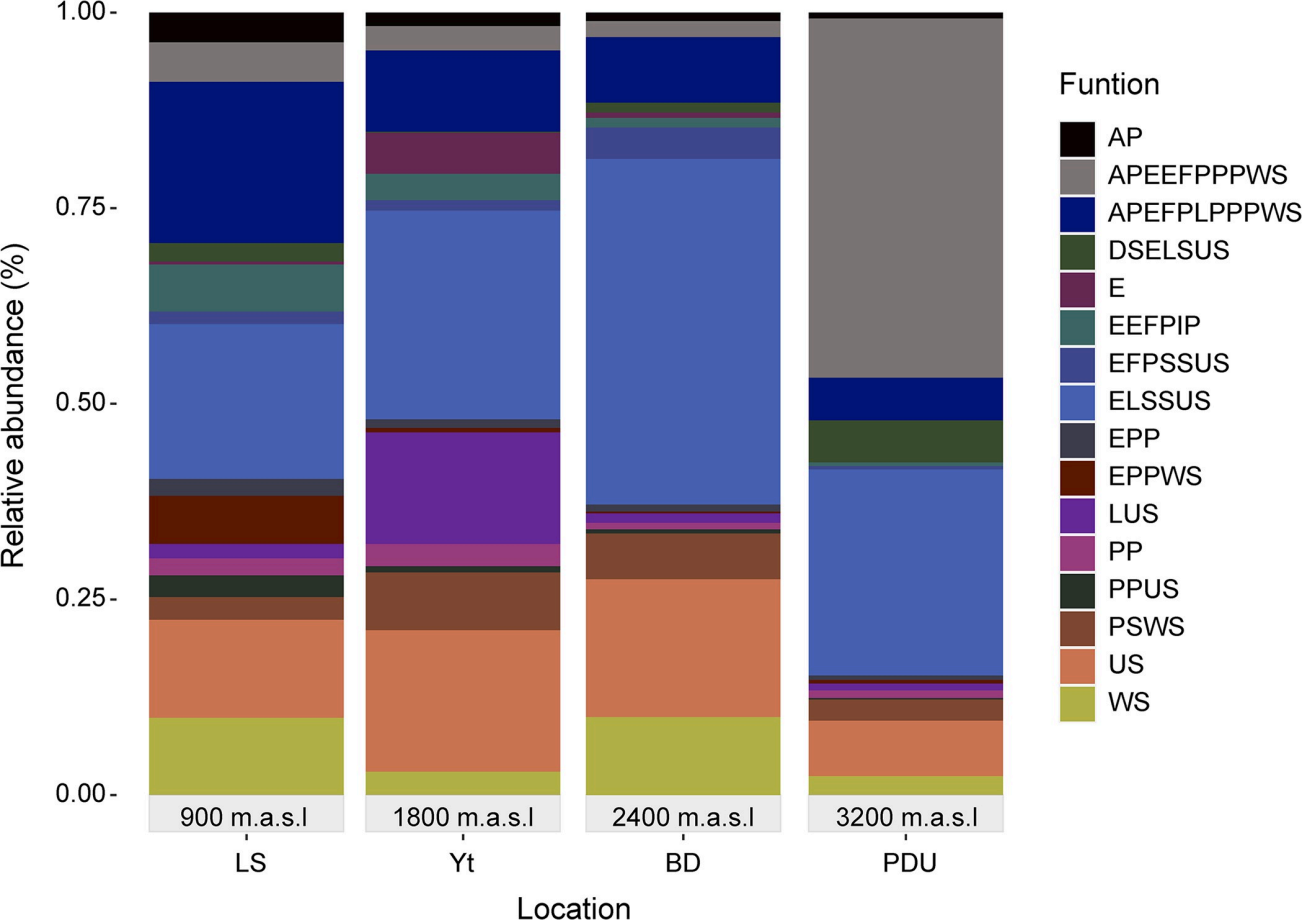
Assignment of functional guilds in fungi for the 4 localities evaluated using FunGuild. (See list of abbreviations S6 Table).

The FUNGuild tool was employed to predict fungal functions based on their ecological guild. This analysis resulted in a 36.5% assignment rate, identifying 97 functional guilds (S6 Table). Notably, 16 of these guilds comprised a substantial portion (85.5%) of the predicted functions and were consistently present across all studied locations (Fig 6 and S6 Table).

FUNGuild identified two dominant fungal functional guilds: endophytes, litter saprotrophs, and undefined soil saprotrophs (ELSSUS), constituting approximately 23.1% of the predicted functions, and animal pathogens, endophytes, fungal parasites, lichen parasites, plant pathogens, and wood saprotrophs (APEFPLPPWS), representing around 12.3%.

The ELSSUS guild was most prevalent in LS and Yt locations and showed a strong association with the abundant genus *Mortierella*. While APEFPLPPWS exhibited a particular preference for LS areas, with notable diversity due to the presence of 27 fungal genera, including *Fusarium* and *Volutella*, suggesting a significant involvement in phytopathogen dynamics and wood decomposition.

Undefined saprotrophs (US) also played a significant role, accounting for approximately 9.7% of the predicted fungal functions and being widely distributed across all studied areas. Genera such as *Leohumicola*, *Podospora*, and *Geastrum* were prominent within this group, suggesting their contribution to organic matter decomposition and nutrient cycling in various environments. Notably, the analysis also identified other guilds with lower representation, such as ECMFPSUS (exclusively associated with *Trichoderma*—5.8%) and those related to ectomycorrhizal activity (ECM—1.6%). While less abundant, their potential roles in soil health and plant symbiosis warrant consideration.

### Modeling and analysis of relationships between variables using PLS-PM

**Fig 7:**
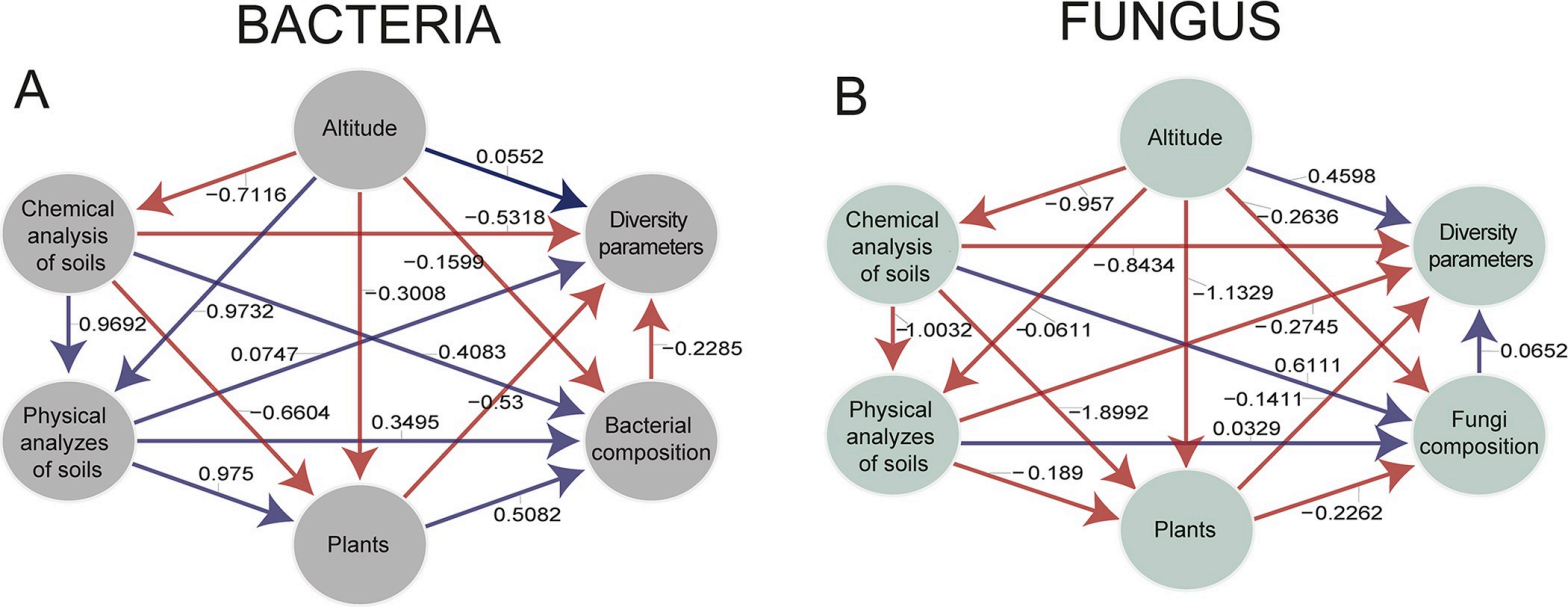
Analysis of relationships between variables using Partial Least Squares Path Modeling (PLS-PM). A: Modeling for Bacteria. B: Modeling for Fungi. Coefficients close to 1 or -1 indicate a strong relationship between the variables and coefficients close to 0 indicate a weak or null relationship. In bold coefficients represent statistical differences with a p-value < 0.05.

The analysis of relationships between variables using PLS-PM revealed interesting patterns for bacteria and fungi (Fig 7). However, a strong negative correlation emerged between altitude vs the concentration of chemical elements in the soil for both bacteria (-0.71), and fungi (-0.95), and this in turn with the diversity of bacteria at different altitudes (-0.53), as well as with fungal diversity (-0.84).

On the other hand, for bacteria a positive influence of chemical elements on bacterial composition (0.40) was observed, being even more pronounced in physical components (0.96), which in turn positively influenced plant composition (0.97); these elements have a moderate influence on bacterial composition (0.34) and no influence on diversity. In the case of fungi, a positive relationship was identified between chemical elements and composition (0.61), while the relationship with physical components was negative (-1), with no apparent influence on plants and fungal composition, but unlike bacteria, in fungi a moderate negative distance coefficient was observed (-0.27) where physical components negatively influence diversity, suggesting that these microorganisms respond differently to environmental conditions.

Regarding plants on bacteria analysis, a significant negative influence of chemical elements (-0.66) and a moderate influence of altitude (-0.30). However, the presence of plants exerts a positive influence on bacterial composition (0.50) but a negative one on bacterial diversity (-0.53). Differential behaviors were observed in fungi, where it was only observed that plants had a moderately negative influence on composition (-0.22).

## Discussion

The study conducted in western Valle del Cauca, Colombia, explored the complex interplay between microbial diversity, altitude, and ecological variables. Considering factors like soil health, plant presence, fungal and bacterial communities, their functional potential, and the altitudinal gradient across four elevations within three distinct ecosystems.

The results showed a high diversity in bacterial composition, with Acidobacteriota (33.1%) and Proteobacteria (23.9%) as the most representative phyla at all studied altitudes. Acidobacteriota, particularly the order Vicinamibacterales, showed variations in abundance along the altitudinal gradient.This bacterial group is associated with the degradation and utilization of available carbon in organic-rich soils, such as those found in this study, as they heavily depend on this source for energy and carbon [19]. Acidobacteriota tend to specialize and are dominant and better adapted to stable and complex communities in environments with high carbon availability [20–22]. Similarly, the presence of Proteobacteria, particularly the genus *Rhizobium*, was associated with important soil conservation functions, such as organic matter decomposition and nitrogen fixation in symbiosis with plants [23], but the most abundant genera found were *Acidibacter*, *MND1*, *Pseudomonas*, and *Candidatus Udaeobacter*, suggesting their contribution to organic matter degradation and the carbon cycle, possibly influenced by the high content of organic matter in all sampled areas.

In terms of fungi, three predominant phyla were identified: Ascomycota (38.6%), Ba-sidiomycota (11.3%), and Mortierellomycota. Ascomycota was the most abundant, with the genus *Archaeorhizomyces* in all locations, particularly in the highest ones (BD and PDU), suggesting a preference for environmental conditions associated with Andean forests and the proximity of these forests to the páramo zone, characterized by high organic matter content, extremely acidic pH, and high available iron content, associated with the symbiotic mycorrhizal role since *Archaeorhizomyces*, like Acidobacteria, can degrade insoluble iron and solubilize it, making it available in the soil [24–26], but this is not completely taken up by forest species due to the negative effect of altitude increase, leading to a lower photosynthetic capacity and a decrease in nutrient use efficiency [27].

Similarly, in the location with the lowest altitude (LS), two genera, *Trichoderma* and *Fusarium*, were identified, showing significant abundance. Despite *Trichoderma* being known for its antagonistic capacity against *Fusarium* in certain circumstances [28, 29], this study revealed a coexistence. *Trichoderma*, recognized for its secondary metabolites with biocontrol agent functions and plant growth promotion [30, 31], does not seem to inhibit *Fusarium* according to the data obtained. This finding suggests a potential interaction between the two genera, indicating that *Fusarium* may be involved in the production of secondary metabolites associated with the decomposition of organic matter [30, 32]. This could have significant implications for the biogeochemical cycles of the local ecosystem, aligning with the broad adaptability and distribution capacity of Ascomycota in various ecosystems [33].

On the other hand, Basidiomycota was mainly associated with the degradation of woody components [34], with the genera *Saitozyma* and *Trechispora* present along with *Trichoderma* in LS. This ecosystem is characterized by sedimentary and floodable soils, suggesting a specific adaptation of these microorganisms to this type of environment. Additionally, Mortierellomycota, represented mainly by the genus *Mortierella*, was the second most abundant group, especially in LS and Yt soils, the lower altitudes. These findings imply that these species may play different ecological roles [35].

In this context, plants contributed to the behavior of microbial composition, where it was found that the Yt location stood out by conserving the highest number of described plant families, with a total of 34, followed by BD with 29, PDU with 20, and LS with 9 families. It was also observed that among the four locations, they do not share any family. However, LS, Yt, and BD share 3 different families: Fabaceae, Salicaceae, and Solanaceae. These findings highlight a dynamic, complex, and multifaceted relationship encompassing symbiosis, competition, and interaction [36].

Consequently, the diversity of bacteria and fungi in the samples was high, with values exceeding 3 in all locations. Although PDU recorded slightly lower values, it is still considered high diversity [37]. However, differences in species abundance were observed between locations, with notable variations in taxonomic classification. Likewise, beta diversity revealed a high number of exclusive taxa in the LS location for bacteria and fungi, decreasing with increasing altitude. Additionally, bacteria showed more abundant assignments at all levels, predominantly in LS in terms of exclusive taxa as well as for fungi. These patterns were supported by PCoA analysis which differentiated samples by location except for BD and PDU which were grouped according to their geographical origin. In addition, the edaphic characteristics of the soil may have contributed to differential shaping of the microbial community structure. Previous studies have demonstrated that factors such as pH, carbon content, effective cation exchange capacity, and organic matter can serve as key determinants of microbial community variations in soil [38–40]. Likewise, physical soil factors such as soil texture and moisture are crucial determinants of bacterial community structure [41, 42]. Conversely, for fungi, physical soil factors did not exhibit a significant impact on diversity, implying a degree of independence of these factors in shaping fungal community composition. Ultimately, a relationship between alpha and beta diversity was found indicating the coexistence of microorganisms with different ecological roles in the different sampling zones [37].

Concerning the above, the analysis of the functional potential of the identified microorganisms revealed a series of relevant functions within the soil microbial ecology. Bacteria showed a significant association between chemo-heterotrophy and the phylum Acidobacteriota, with genera like *Edaphobacter*, *Acidicapsa*, *Candidatus Koribacter*, and *Granulicella* predominant in the highest altitude location (PDU). This relationship between chemo-heterotrophy and the nitrogen cycle is one of the most reported in other studies with similar patterns in the Brazilian Amazon [43, 44], evidenced in this research by the presence of nitrifying bacteria such as *Nitrosomonas* and *Nitrobacter*, albeit in low abundances.

Considering the above, it is noteworthy that there is an indirect influence of the altitudinal gradient, where chemical elements in the soil decrease as altitude increases. This trend is associated with lower average annual temperatures, higher soil moisture, and high concentrations of phenolic compounds that slow down the decomposition and mineralization of organic matter at higher altitudinal gradients [27].

On the other hand, the relationship between plant presence and microbial composition also showed interesting patterns. A positive association was found between plant presence and bacterial composition, but a negative association with fungal diversity. Similar results were reported in a study on tropical oligotrophic swamp soils [45] highlighting the complexity of interactions among soil microorganisms, soil properties, and vegetation in the studied ecosystems. Additionally, they suggest that additional environmental factors, such as altitude and the concentration of chemical elements in the soil, can significantly modulate these interactions.

## Materials and methods

### Study area

**Fig 8:**
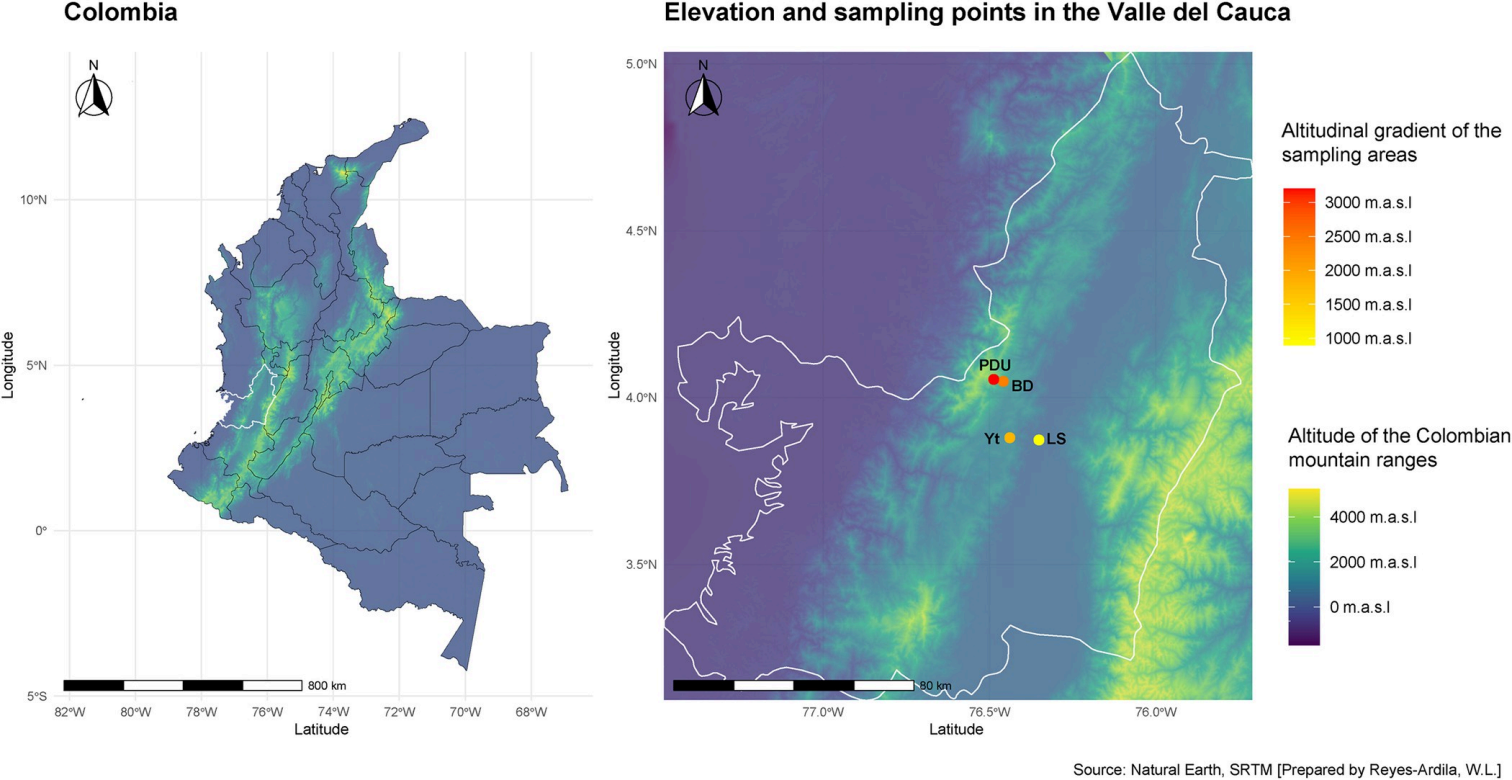
Distribution of geographical altitudes of the locations and ecosystems studied (LS: Laguna de Sonso, Yt: Bosque de Yotoco, BD: Bosque del Duende, PDU: Páramo del Duende).

Between September and December 2021, soil samples were collected from forested areas across an altitudinal gradient in the western range of the Andes, Valle del Cauca, Colombia (Fig 8). Sampling commenced at Laguna de Sonso (LS) located at 900 m.a.s.l. within the Tropical Dry Forest, followed by Yotoco Forest Reserve (Yt) at 1,800 m.a.s.l., Bosque del Duende (BD) at 2,400 m.a.s.l., and culminating at Páramo del Duende (PDU) at 3,200 m.a.s.l. Notably, both Bosque del Duende and Páramo del Duende are situated within the Parque Natural Regional Páramo del Duende.

### Soil sampling along an altitudinal gradient

To ensure the representativeness of the soil samples, we were collected at a depth of 0 to 25 cm in all locations, removing the litter. In most areas, three plots of 10 x 10 m were established, and three individual samples were obtained from each plot. However, due to the distinct topographical characteristics of the PDU, a paramo, ecosystem with a single access road bordered by steep cliffs, only two plots were designated, but five samples were collected from each, resulting in a total of 37 samples for physicochemical analysis and microbial DNA extraction. Additionally, flora characterization was conducted in the field for each of the evaluated plots. The map (Fig 8) was generated utilizing the rnaturalearth R package, which employs the publicly available Natural Earth geospatial data set.

### Physicochemical analysis

From the sampling process described earlier, specific samples were collected to analyze the soil’s physicochemical components. Approximately 1 kg of soil was taken from each of the 37 samples. These samples were analyzed for their chemical fertility by the Corporación Colombiana de Investigación Agropecuaria–AGROSAVIA, an accredited laboratory under ISO/IEC 17025:2017. The evaluation included elements such as soil organic matter (SOM), soil organic carbon (SOC), available phosphorus (P), pH, exchangeable calcium (Ca), exchangeable magnesium (Mg), ex-changeable potassium (K), exchangeable sodium (Na), effective cation exchange capacity (ECEC), available nitrogen (N), available iron (Fe), available copper (Cu), available manganese (Mn), available zinc (Zn), available boron (B), electrical conductivity (EC), and sulfur (S). According to the results provided by AGROSAVIA, the following standardized protocols were used: GA-R-46 for pH, NTC 5526:2007 for micronutrients, GA-R-50 for exchangeable bases, GA-R-119 for organic carbon determination, and NTC 5596:2008 for electrical conductivity. Additionally, bulk density (BD) was analyzed using the cylinder method [46, 47] and soil texture was assessed using the Bouyoucos method [48]. The collected data underwent descriptive statistical analysis, followed by a Shapiro-Wilk normality test and a non-parametric Kruskal-Wallis, and Dwass-Steel-Critchlow-Fligner pairwise comparisons. All these analyses were performed using Jamovi 2.2.5 Solid®.

### DNA extraction and sequencing

The 37 soil samples were individually taken and stored in 25 ml Falcon tubes. These were refrigerated and transported to the molecular biology laboratory at the Universidad Nacional de Colombia, Palmira campus. There, they were stored at -80°C to preserve the integrity of the DNA. The genetic material was extracted using the DNeasy PowerSoil Pro Kit (Qiagen, Hilden, Germany) following the manufacturer’s instructions.

To assess the quality of the extracted DNA, we employed a Colibri Titertek Berthold 84030 spectrophotometer and analyzed samples on 0.8% agarose gels. The V3-V4 regions of the 16S rRNA gene for bacteria were amplified using primers 341F (CCTAYGGGRBGCASCAG) and 805R GGACTAC-NNGGGTATCTAAT) [49], while ITS regions for fungi were amplified using primers ITS5-1737 (GGAAGTAAAAGTCGTAACAAGG) and ITS2-2043R (GCTGCGTTCTTCATCGATGC) [50]. This process yielded results for both bacteria and fungi groups from the 37 samples each. Sequencing was performed using the Illumina Novaseq 6000 system, generating ~150,000 reads per sample. Finally, the sequences from 74 samples were deposited in the European Nucleotide Archive (ENA) under project number PRJEB61162 with specified accessions (S1 Table).

### Bioinformatic analysis

We initiated a data sequencing quality control using DADA2 in QIIME2 v.2022.2 [51]. This involved removing chimeric sequences and identifying Amplicon Sequence Variants (ASVs). Subsequently, the SILVA v13_8 and UNITE v8-99 databases were utilized for taxonomic assignment in bacteria and fungi, respectively. Statistical analyses were conducted in R Studio v.4.2.2 with the QIIME2R, and phyloseq packages [52]. Graphs depicting the most abundant taxa were generated using ggplot2 [53].

Furthermore, for alpha diversity, Shannon, Pielou, Berger-Parker indices, and abundance-based rarity index were calculated using the microbiome package. Regarding beta diversity, Venn diagrams and PCoA analysis based on Bray-Curtis dissimilarity were employed using the packages Venn and Phyloseq respectively. Subsequently, variations in microbial group abundance were identified using DESeq2 [54], and relationships between variables were established a multiple factorial analysis (MFA) plot using the FactoMineR package [55].

The functional potential of bacteria and fungi was assessed with FAPROTAX [56] and FUNGuild [57], respectively. Most abundant potential annotations were visualized in a heatmap and PCA for bacteria and a bar graph for the guilds for fungi, using the ggplot2 package. Finally, a Partial Least Squares Path Modeling (PLS-PM) multivariate data modeling analysis was implemented to determine relationships between microbial communities, alpha diversity, and environmental factors through the plspm package.

## Conclusions

This study in the western Andes in the Valle del Cauca, Colombia, focuses on the interactions among microorganisms, environmental variables, and altitudinal gradients in three strategic ecosystems. The results reveal distinctive patterns in bacterial and fungal composition, with Acidobacteriota and Proteobacteria as the most representative phyla, highlighting their potential role in carbon cycle and soil conservation. The high diversity of fungi, especially Ascomycota, suggests significant implications for biogeochemical cycles. The coexistence of *Trichoderma* and *Fusarium* indicates a symbiotic interaction with effects on organic matter decomposition. Additionally, an indirect influence of the altitudinal gradient on microbial diversity was found, along with a direct association between plant presence and microbial composition, emphasizing the complexity of these interactions in the studied ecosystems.

## Supporting information

S1 Table Sequence quality and information in the European Nucleotide Archive. (XLSX)

S2 Table ASVs information. (XLSX)

S3 Table Plant Information and abbreviations implemented in the MFA. (XLSX)

S4 Table Bacterial function data. (CSV)

S5 Table Fungal functions data. (CSV)

S6 Table Contribution of MFA analysis dimensions. (XLSX)

S7 Table Abbreviations of fungal functions. (XLSX)

S1 Fig Bacteria Deseq. (TIF)

S2 Fig Fungal Deseq. (TIF)

S3 Fig PCA of bacterial functions. (TIF)
