# Supplementary figures and images for: Exploring soil bacterial and fungal communities in Colombian terrestrial ecosystems modulated by altitude-influenced factors
Source: PLoS One. 2024 Dec 12;19(12):e0312842. doi: 10.1371/journal.pone.0312842 (PMC11637269; doi:10.1371/journal.pone.0312842)

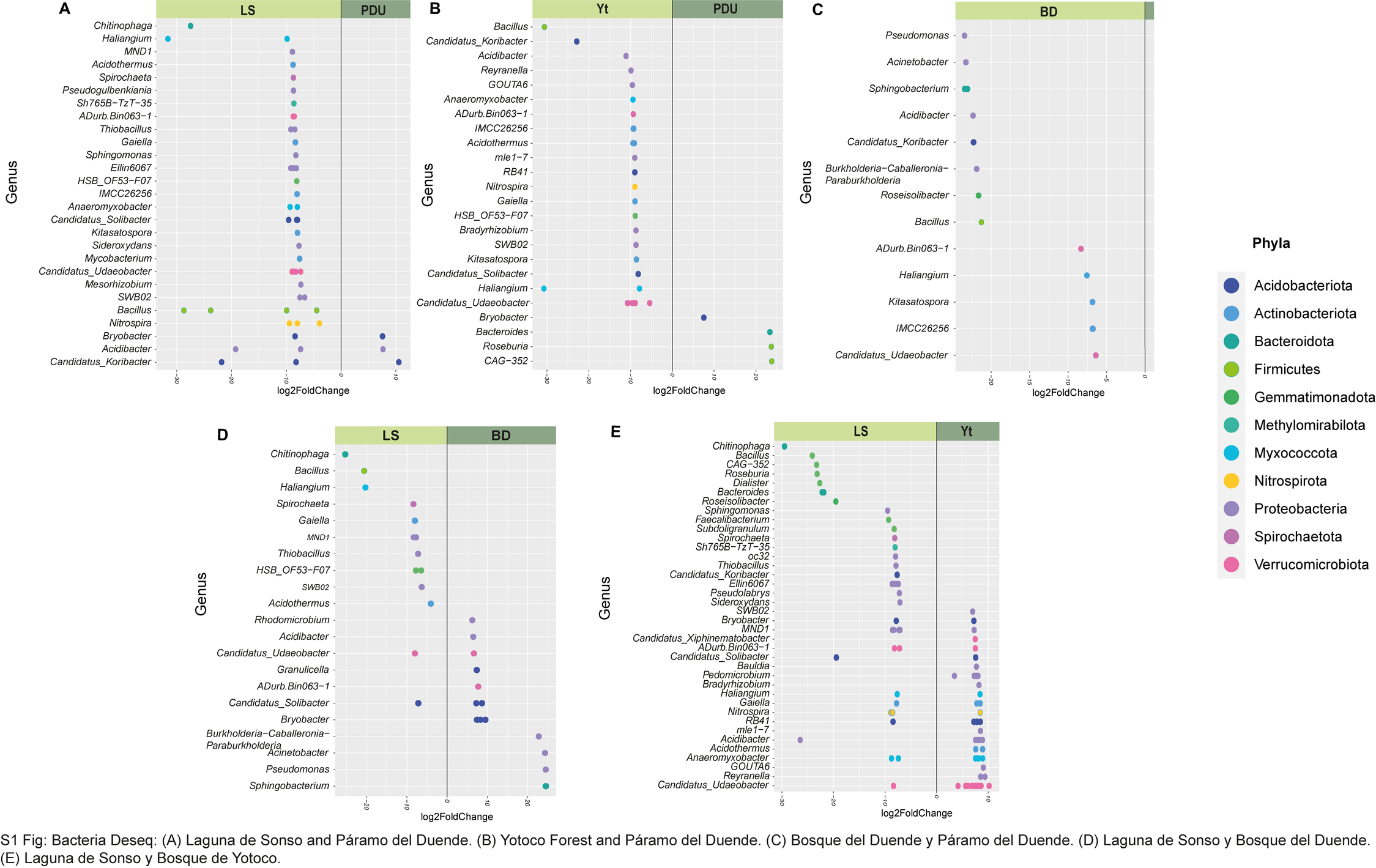

Supplement: S1 Fig — (TIF) [file pone.0312842.s008.tif]

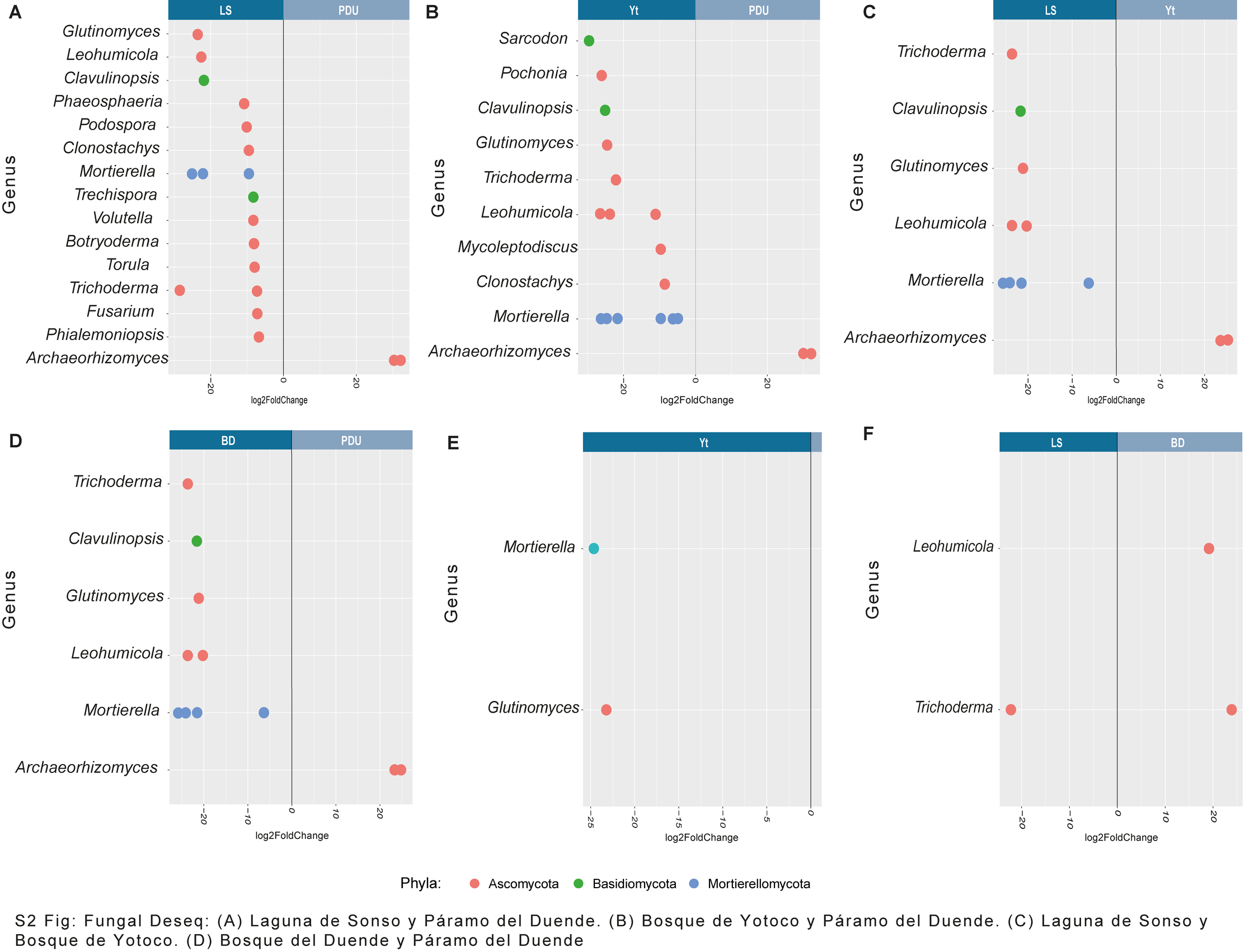

Supplement: S2 Fig — (TIF) [file pone.0312842.s009.tif]

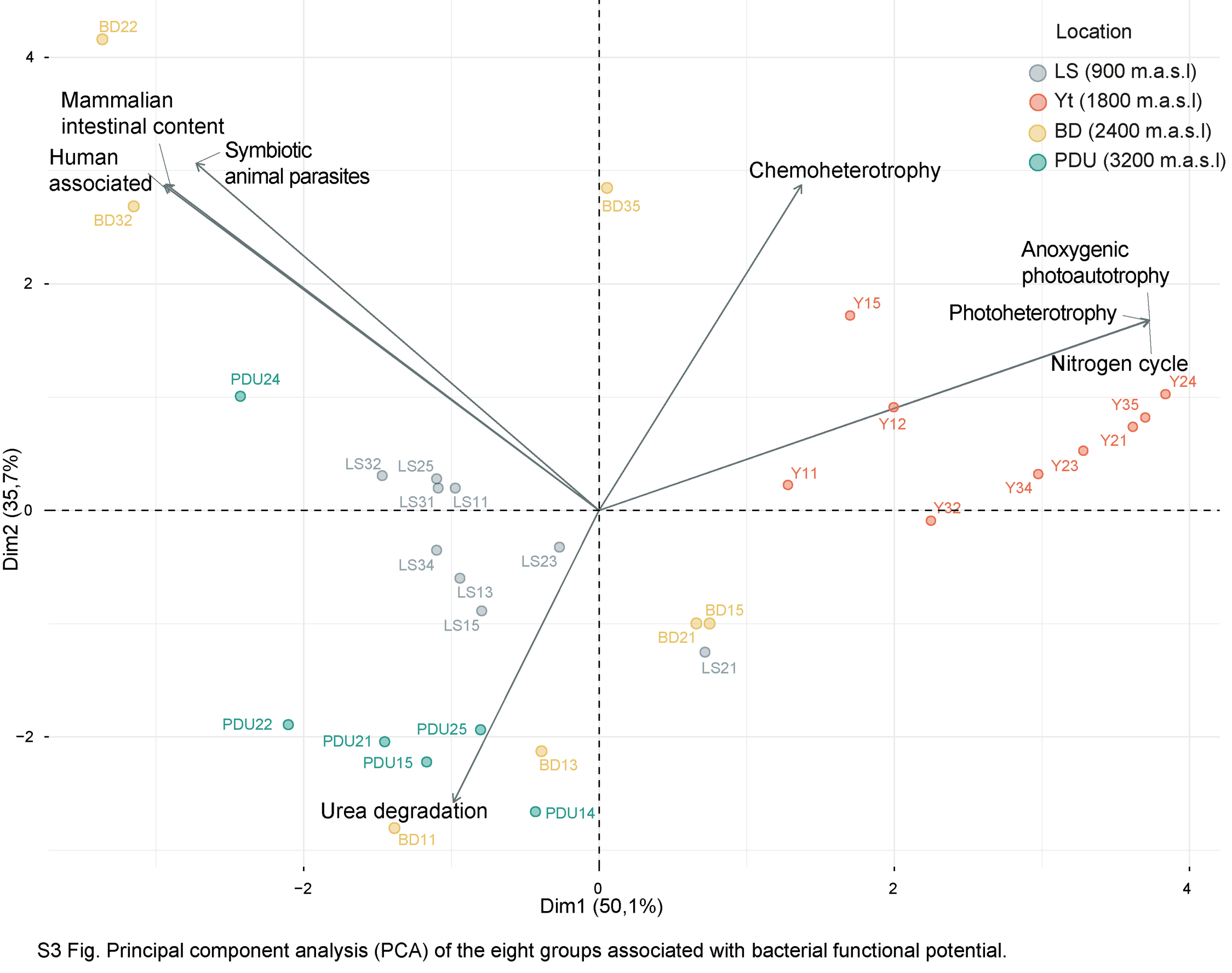

Supplement: S3 Fig — (TIF) [file pone.0312842.s010.tif]
